# Simultaneous Determination of Phenolic Compounds in *Leptadenia pyrotechnica* (Forssk.) Decne. by Using High-Performance Liquid Chromatography (HPLC-DAD-UV)

**DOI:** 10.1155/2018/9604972

**Published:** 2018-10-21

**Authors:** Raman Preet, Raghbir Chand Gupta

**Affiliations:** Department of Botany, Punjabi University, Patiala 147002, Punjab, India

## Abstract

During the present study, an endeavor has been made to produce a simple, rapid, and simultaneous method for determination of phenolic compounds by using high-performance liquid chromatography in aerial parts of *Leptadenia pyrotechnica* (Forssk.) Decne. collected from the Indian Thar Desert. The optimized process was used for the quantification of ten phenolic compounds. The chromatographic separation was accomplished on an Atlantis T3 column at 25°C with isocratic elution. A mixture of acetonitrile and water was used as the mobile phase at a flow rate of 0.8 mL/min. The linear regression examination data for the calibration plots displayed a good linear relationship with *r*^2^ > 0.999 in the concentration range of 2–20 *µ*L. In the methanolic extracts of the whole plant of *L. pyrotechnica*, the content of caffeic acid (3.3%) was reported to be the highest concentration.

## 1. Introduction

*L. pyrotechnica* is a characteristic desert plant [1] commonly known as Khimp, Kip, or Kheep [2]. It is an erect ascending, leafless, sappy, evergreen shrub which grows from 1.5 m to 3 m high. The stem is glabrous, pale yellow to light green in color and excretes watery fluid or sap. Flowers are bisexual, pentamarous, actinomorphic, and yellowish green in color. Flowering and fruiting of the plant occurs during the months of August to January. It is native to semiarid deserts of African and Asian countries. In India, it is found in dry sandy soil area, and in western India, especially in Rajasthan, it is found with less frequency in Gujarat and Punjab.

It is a wonderful desert plant, of which almost every plant part is employed in the traditional medicinal system to treat several ailments [3]. This species embraces diversity of bioactive ingredients that generate therapeutic properties; it has also been reported to possess the antitumor and anticancer activity. This plant species holds antifungal, antibacterial, anticancer, antioxidant, anthelmintic, antiatherosclerotic, antidiabetic, wound-healing, and hepatoprotective activities [4, 5]. Nearly every plant parts are used in the therapeutic system for the treatment of various disorders. It is distributed throughout the desert habitats of the state [2]. This plant species is an important component of an arid ecosystem and a good source of medicines, forage, and fiber. It is one of the botanical sources of the Ayurvedic drug *Jivanti*. *Jivanti* is one of the important *rasayana* drugs in Ayurveda. It is used as an ingredient in formulations like *Jivantadya taila*, *J. rasa*, *J. ghrita*, *Ashwagandhadi ghritha*, *Anuthaila*, and *Chandanadi thaila*. These formulations are effective in the treatment of diseases like hemorrhage, tuberculosis, emaciation, fever, and cardiac ailments [6, 7]. Earlier, HPLC studies also showed the high concentration of *β*-sitosterol, lupeol, and oleanolic acid in *L. pyrotechnica* [8]. The plant is one of the ingredients of many formulations which have been used to recover from physiological, bacterial diseases, or even from cancer. The goal of this paper is to analyze qualitatively and quantitatively the phenolic compounds in *L. pyrotechnica*, a highly important medicinal plant of the Indian Thar Desert.

## 2. Experimentation

### 2.1. Sample Collection and Preparation

Fresh healthy aerial plant parts were collected from Rajasthan. Plants were shade-dried, crushed into fine powder, accurately weighed, and exhaustively extracted using methanol. Methanolic extractions were carried out by using Soxhlet apparatus. Samples were clarified by nylon 6, 6 ultipore film filter papers through a pore size of 0.22 *µ*m prior to giving the injection.

### 2.2. Chemicals and Reagents

HPLC-grade methanol, acetic acid, water, and acetonitrile were obtained from MERCK, Mumbai. Ten phenolic standards were purchased from Himedia, India. The stock solution was made by using methanol. The standard calibration curves have high degrees of linearity (*r*^2^ > 0.999). The plant extracts were recognized from the source of the retention of the standard, and quantification was carried out by equating the peak areas with the standards.

### 2.3. Instrumentation and Apparatus

Compounds were investigated by using high-performance liquid chromatography, and simultaneous determination of the phenolic compounds was performed. The separation process was performed on an Atlantis T3 column (5 *µ*m pore size; 4.6 mm × 250 mm) with a diode-array detector held at 25°C. Determination of analytes was carried out on the peak area. Spectra were obtained at 200–600 nm. The developed method has been applied to the plant extracts.

### 2.4. Chromatographic Conditions

The mobile phase was purified using acetonitrile and water with 0.1% acetic acid. The mobile phase was filtered with 5 *µ*m pore filters and then, sonicated in PCI analytics for degassing. Prior to running the baseline of the HPLC-DAD system, the mobile phase was monitored until it became stable before sample analysis.

### 2.5. Method Development

The method has been effectively applied to the determination of phenolic compounds. HPLC-DAD separation of phenolic acids was accomplished according to previous methods with little alterations [9]. The present study is carried out to study the diversity of phenolic acids in methanolic extracts of this plant species.

Acetonitrile is preferred in method development as it provides lesser viscosity, lower pressure, and superior peak regularity of phenolic compounds. Initially, a gradient run from 2% to 92% was achieved to determine whether isocratic or gradient elution was appropriate [9]. In most of the cases, gradient elution is preferred but during the present study, satisfactory separation was achieved by isocratic elution, to decrease analysis time and enhance the separation selectively. Subsequently, the separation was performed on isocratic elution.

The phenolic compounds have the ionizable carboxyl group in their structures; hence, pH has critical significance in separation of these compounds. A mobile phase of lower pH using acetic acid (2-3) was used, and it was buffered to constrain the ionization of analytes and increase column efficiency. The separation of overlapping peaks and restriction of peak tailing of caffeic acid and resorcinol of phenolic compounds was achieved at pH 3. The peak tailing might occur due to the interface between partially ionized analyte and surface column. The separation of all the peaks with higher resolution was found at 23°C. The consequence of the flow rate on separation was discovered by altering the flow rate from 0.5 to 1.5 mL/min. By reducing the flow rate, a proportional increase in the analysis time was obtained in order to sustain peak spacing. The analysis flow rate of the mobile phase was preserved at 0.8 mL/min, and adequate separation was achieved.

**Figure 1:**
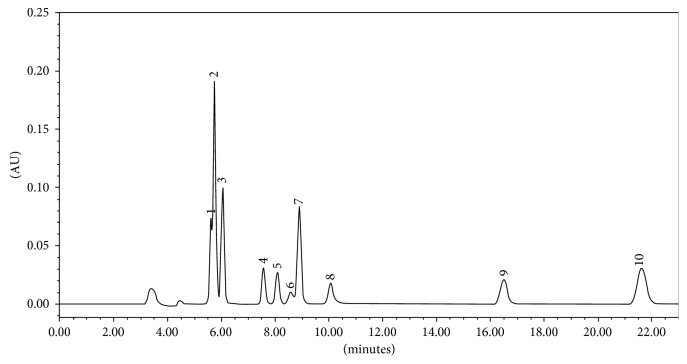
A typical HPLC chromatograph showing the standard mixture of phenolic compounds, i.e., [1] caffeic acid, [2] resorcinol, [6] vanillic acid, [7] *p*-coumaric acid, [9] ferulic acid, [10] vanillin, [11] veratric acid, [4] myristicin, [5] coumarin, and cinnamic acid.

These optimal chromatographic conditions were employed to resolve the phenolic compounds in 22 min. All the peaks were baseline-separated (>1.5) with the resolution in 22 min.  Figure 1 shows a characteristic chromatogram of 10 *µ*g/mL standard blend of phenolic acids using the optimized conditions. Among the ten phenolic compounds, caffeic acid (3.30%) was reported in maximum amount (Table 1).

### 2.6. Validation

These studies resembling linearity range, precision, accuracy, limit of detection, and limit of qualification have also accomplished to recognize that the formed method was appropriate for practical examination in agreement with the guidelines of the International Conference on Harmonization [10]. The method linearity has been assessed by plotting the peak area versus the concentration of the analytes from 2.0 to 20 *µ*g/mL. Calibration curves were obtained by three sequential quantities of the five concentrations. All the correlation coefficients achieved indicate that the method displayed excellent linearity within the studied range.

The limits of detection and limits of quantification were calculated using the following equations: LOD = 3.3*s*/*m* and LOQ = 10*s*/*m*, employing the standard deviation of *y*-intercept (*s*) and the slope (*m*) of the corresponding calibration curve [11]. The consequences demonstrated that the mentioned method delivers suitable linearity, sensitivity, accuracy, and precision for the synchronized analysis of phenolic compounds in any plant metrics.

## 3. Results and Discussion

**Figure 2:**
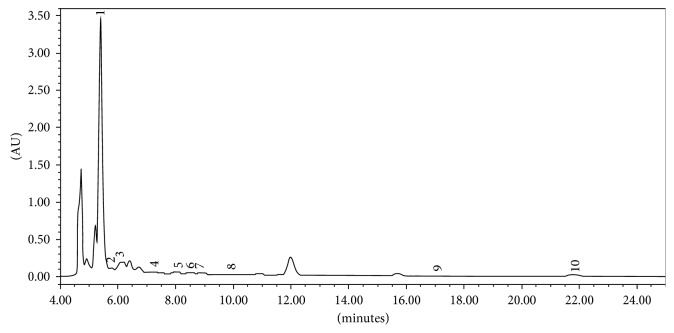
A typical HPLC chromatograph showing phenolic compounds in aerial parts of *Leptadenia pyrotechnica*.

*L. pyrotechnica* is considered an important medicinal plant and is widely used by local/tribal people of the Indian Thar Desert. The objective of this study was to produce a technique for simultaneous separation of phenolic compounds from a crude methanolic extract by means of HPLC. To obtain a more comprehensive profile of these phenolic compounds, a simple, rapid, analytical HPLC method was developed, using acetonitrile and water as the mobile phase (Figure 1). As a result, it was found that it contains the highest concentration of caffeic acid (3.3%) followed by vanillin (0.018%) (Figure 2). Table 1 demonstrates the concentration of entirely studied phenolic compounds, and Table 2 shows validation parameters of the liquid chromatographic method. Previously, there were many reports of composition of various phytochemicals in this plant species, but the present study is the first attempt to develop a simultaneous method of determination of phenols in crude plant matrices.

## 4. Conclusion

An efficient, rapid, simple method for simultaneous separation of phenolic compounds is developed. The method is suitable for obtaining a comprehensive HPLC profile. The respective compounds were identified by peak areas and *R*_*f*_ values. The content/concentration was calculated by calibration curves. The presence of caffeic acid in the highest concentration followed by other compounds in aerial parts of this desert plant indicates its rich phytochemical composition and its potential to be used in drug and pharmaceutical companies and hence proves its wide usage by local/tribal people for various alignments.

## Figures and Tables

**Table 1:** Data showing the amount of ten phenols in aerial parts of *L. pyrotechnica*.

S. no.	Phenolic compound/flavonoid	Amount (%)
1	Caffeic acid [1]	3.305
2	Resorcinol [2]	0.008
3	Vanillic acid [6]	0.063
4	p-Coumaric acid [7]	0.034
5	Ferulic acid [9]	0.054
6	Vanillin [10]	0.181
7	Veratric acid [11]	0.015
8	Myristicin [4]	0.013
9	Coumarin [5]	0.004
10	Cinnamic acid	0.042

**Table 2:** Validation of the liquid chromatographic method.

S. no.	Analyte	Retention time	Concentration range (*µ*g/mL)	Calibration equation	Correlation coefficient	Limit of detection (*µ*g/mL)	Limit of quantification (*µ*g/mL)
1	Caffeic acid [1]	5.616	1–20	*Y* = 8.20*e* + 004*X* − 4.64*e* + 004	0.999836	0.22	0.63
2	Resorcinol [2]	5.745	1–20	*Y* = 3.66*e* + 004*X* − 1.03*e* + 004	1.000000	0.28	0.66
3	Vanillic acid [6]	6.050	1–20	*Y* = 1.10*e* + 005*X* − 5.72*e* + 004	0.999948	0.22	0.70
4	*p*-Coumaric acid [7]	7.563	1–20	*Y* = 1.88*e* + 004*X* − 8.97*e* + 003	0.999995	0.21	0.66
5	Ferulic acid [9]	8.084	1–20	*Y* = 1.97*e* + 004*X* − 2.17*e* + 004	0.999669	0.28	0.75
6	Vanillin [10]	8.575	1–20	*Y* = 1.59*e* + 004*X* − 8.61*e* + 003	0.999997	0.21	0.65
7	Veratric acid [11]	8.899	1–20	*Y* = 6.32*e* + 004*X* − 3.56*e* + 004	0.999940	0.22	0.71
8	Myristicin [4]	10.061	1–20	*Y* = 1.14*e*+ 0 04*X* − 2.36*e* + 004	0.997837	0.25	0.72
9	Coumarin [5]	16.501	1–20	*Y* = 1.67*e* + 005*X* + 2.60*e* + 004	0.999182	0.22	0.69
10	Cinnamic acid	21.630	1–20	*Y* = 7.73*e* + 004*X* − 4.34*e* + 004	0.999998	0.23	0.67

## Data Availability

The data used to support the findings of this study are available from the corresponding author upon request.
